# Infectious agents in juvenile scleroderma

**DOI:** 10.1186/1546-0096-12-S1-P306

**Published:** 2014-09-17

**Authors:** Iryna Chyzheuskaya, Lyudmila Belyaeva, Rostislav Filonovich, Larisa Zaitseva, Alexander Lyatun

**Affiliations:** 1 Pediatrics, Belarusian Medical Academy of Postgraduate Education, Minsk, Belarus; 2 4th City Children's Hospital, Minsk, Belarus; 3 belarusian Medical Academy of Postgraduate Education, Minsk, Belarus

## Introduction

The problem of juvenile scleroderma (JS) is determined by a variety of clinical manifestations, tendency to early generalized process with the development of peripheral and visceral lesions. In the last decade various forms JS associated with Borrelia infection, as evidenced by the discovery of a skin biopsy patients spirochete Borrelia Burgdorferii afzelii, garinii and the presence of specific antibodies in the blood of patients. Besides bacterial relevant theory and molecular "mimicry" the herpes viruses, providing a provocative role in the development of JS.

## Objectives

To determine the role of infectious factors in the genesis of juvenile scleroderma.

## Methods

65 children with juvenile scleroderma were examined. Patients were divided into 2 groups: 41 patients with scleroderma (S) (mean age 13,1±0,4 years) and 24 with systemic sclerosis (SS) (mean age 12,7±0,5 years). All patients underwent bacteriological examination with nasopharyngeal flora definition of sensitivity to antibiotics. Antibodies in the blood was determined by Borrelia burgdorferi, Chlamydia psittaci, Herpes Simplex Virus Types 1, 2 and Cytomegalovirus. The concentration of interferon-gamma (IFN-γ) in the serum was determined by ELISA using kits from Immunotech (France).

## Results

The most of children with the S (56.1%) and SS (87.5%) had chronic foci of infection (chronic tonsillitis, chronic pharyngitis, adenoids, chronic periodontitis). Staphylococcus aureus was allocated from the nasopharynx in 19.5% of patients with S and 25% of children with SS, β-hemolytic streptococcus was allocated from 17.1% of children with S and in 30.8% of the SS. Antibodies to Borrelia burgdorferi in blood serum identified in 39.1% of children with S and 37.5% of children with SS. Antibodies to Chlamydia psittaci in blood serum identified in 12.2% of patients with S and 16.7% of the SS.

The presence of chronic CMV infection revealed in 17% of children with the S and 20.8% of the SS. The presence of the herpes simplex virus infection revealed in 14.6% of patients with S and 25% with SS. Level of IFN-γ in serum was significantly lower in all children with S and SS than in healthy children (0,76±0,2 pg/ml). Reduction of IFN-γ plays a role in reducing the antiviral immunity and confirmed the role of RNA viruses in the development and progression of juvenile scleroderma.

## Conclusion

The foci of chronic infection alter the reactivity of the organism causing an imbalance in the immune system of children, and is likely to play a pathogenetic role in the occurrence of juvenile scleroderma. The presence of persistent viral infection and a significant decrease of IFN-γ in children with SS and S suggests a role of viral infection in the development and progression of juvenile scleroderma.

## Disclosure of interest

None declared.

